# Radiotherapy improves stress urinary incontinence but impairs pelvic floor function in endometrial cancer patients: a prospective cohort study

**DOI:** 10.1007/s00404-025-07964-1

**Published:** 2025-01-30

**Authors:** Selçuk Erkılınç, Ilker Çakır, Volkan Karataşlı, Behzat Can, Can Ata, Aytuğ Avşar, Ulaş Solmaz, Muzaffer Sancı, Tevfik Berk Bildacı

**Affiliations:** 1 Department of Gynecologic Oncology, İzmir Democracy University School of Medicine, Buca Seyfi Demirsoy Education and Research Hospital, İzmir, Turkey; 2 Department of Gynecologic Oncology, Balikesir City Hospital, Balikesir, Turkey; 3 Department of Gynecologic Oncology, Diyarbakir Gazi Yaşargil Education and Research Hospital, Diyarbakir, Turkey; 4 Gynecologic Oncology, Private Clinic, İzmir, Turkey; 5 University of Health Sciences İzmir City Hospital, İzmir, Turkey

**Keywords:** Radiation therapy in endometrial cancer, Urinary incontinence, Endometrial cancer

## Abstract

**Purpose:**

Investigating the impact of radiotherapy on urinary incontinence and pelvic floor dysfunction in endometrial cancer patients.

**Method:**

A comparative study was conducted between endometrial cancer patients who underwent radiotherapy and those who did not receive adjuvant therapy. Patients were assessed during their first follow-up visit at third month post-radiotherapy or post-surgery. Demographic data and physical examinations were conducted, along with the administration of validated questionnaires. Turkish validated Incontinence Severity Index (ISI), Incontinence Impact Questionairre-7 (IIQ-7) and 20 ıtem Pelvic Floor Dysfunction Index (PFDI-20) were applied to the all patients.

**Results:**

The study comprised 37 patients in the non-radiotherapy group and 41 patients in the radiotherapy group. Comparable demographics were observed between the two groups. Vaginal length was notably longer in the non-radiotherapy group, and the Q-tip test angle was significantly greater in this group. A higher incidence of stress urinary incontinence and higher scores on the Incontinence Severity Index were noted in the non-radiotherapy group. Conversely, the radiotherapy group exhibited significantly higher scores on the Pelvic Floor Dysfunction Index components and total score. Urogenital Distress Inventory scores were similar between the groups.

**Conclusion:**

Radiotherapy showed mixed effects on pelvic floor function in endometrial cancer patients. While it potentially improved stress urinary incontinence, it was associated with unfavorable outcomes in overall pelvic floor dysfunction.

## What does this study add to the clinical work

**Table:** 

This study provides specific and quantifiable data on the effects of radiotherapy on urinary incontinence and pelvic floor dysfunction in endometrial cancer patients. Using objective measures such as the Q-tip test, ISI, IIQ7, CRADI-8, UDI-6, and POPDI, we were able to assess the impact of radiotherapy with quantifiable measures.	

## Introduction

Although it is frequently observed, most of endometrial cancer patients are diagnosed in earlier stages than those of other gynecologic cancers [1]. The treatment landscape for endometrial cancer is evolving toward more limited surgical approaches, with conservative methods such as sentinel lymph-node mapping being accepted as the standard for reducing surgical morbidities globally [2]. Moreover, the publication of molecular evaluations and staging based on molecular features has shifted clinicians’ attention to endometrial cancer [3]. Indicators suggest a growing trend toward more conservative approaches for managing endometrial cancer. In addition to the extent of surgery, the type of surgery may also affect pelvic floor dysfunction. However, both open and laparoscopic surgeries have been shown to have similar pelvic floor dysfunction outcomes [4]. External radiotherapy as an adjuvant therapy for high-intermediate and high-risk patients, has been reported to be overused [5]. Technological advancements have reduced radiotherapy-associated Grade II and Grade III toxicity in endometrial cancer patients [6]. Nonetheless, women treated with RT (Radiotherapy) have reported compromised quality of life, including irritation cystitis, stress urinary incontinence, and abnormal voiding [7]. While most related studies have focused on the effects of radiotherapy on cervical cancer, limited research has investigated the impact of radiotherapy on endometrial cancer patients. Therefore, in this study, we hypothesized that radiotherapy would have more adverse effects on urinary incontinence and impaired pelvic floor function in endometrial cancer patients.

As far as we know, specific data regarding pelvic floor abnormalities in patients with endometrial cancer is currently unavailable. Furthermore, there has been no study evaluating the impact radiotherapy on pelvic floor dysfunction and urinary incontinence. The objective of this study was to adress the impact of radiotherapy using urogynecologic evaluation including vaginal length Q-tip test and the inventories including Pelvic Floor Dysfunction Inventory, Urogenital Distress Inventory, and Incontinence Impact Questionairre-7 in endometrial cancer patients.

## Methods

Following approval from the institutional review board (Tepecik Education and Research Hospital-2018/2–22), this study was carried out at a tertiary referral center for gynecologic oncology between January 2018 and January 2019. During the study period, a total of 124 endometrial cancer surgeries were performed. Twenty-six of these patients were excluded from the study because they were in advanced stages. Thirteen patients were excluded from the study because they had undergone prior vaginal surgery for incontinence, or were using medication for overactive bladder were excluded. Seven patients were not included in the study due to lack of follow-up. A total of 78 patients, including 41 who did not receive adjuvant therapy and 37 who received adjuvant therapy, were included in the study. The decision on adjuvant radiotherapy was made by multidisciplanry team including gynecologic oncologists and radiation oncologists. Patients in high-Intermediate risk administered adjuvant radiotherapy. A complete urine test was conducted to detect urinary tract infections, and patients showing signs of infection were treated for urinary tract infection.

Patients who received radiotherapy were assigned to the radiotherapy group, and radiotherapy was administered 7–8 weeks after surgery. Patients in the radiotherapy and no- radiotherapy groups were included to the study during their initial follow-up visit which was three months after completing treatment. After the patients provided informed consent, a regular medical history was taken, and all patients underwent a gynecologic evaluation. Vulvar abnormalities were noted, and assessments for cystocele, rectocele, vaginal cuff descent, or prolapse were conducted. Vaginal length was measured using a disposable wooden stick, and the distance between the vaginal introitus and the vaginal cuff was considered as vaginal length. A cough stress test was conducted while the bladder was full, and the bladder volume was confirmed using ultrasonography. The cough test was applied in both the dorsolithotomy and standing positions. Any urinary leakage was considered positive for stress urinary incontinence. For the Q-tip test, a cotton swab was inserted into the external urethral opening, and patients were instructed to cough. The angle between the neutral position and the final position after the cough stress test was evaluated. All patients completed a validated Turkish Incontinence Impact Questionnaire [8]. All patients underwent administration of the Incontinence Severity Index which evaluates the frequency and quantity of urine loss [9]. The Pelvic Floor Distress Inventory-Turkish validated short form-20, which included three subcategories, namely the urinary distress inventory, colorectal anal distress inventory, and pelvic organ prolapse distress inventory, was utilized [10]. Each subcategory included four answers, with 0 representing no complaints and 4 representing the highest intensity of patient complaints. In accordance with the journal’s guidelines, we will provide our data for independent analysis by a selected team by the Editorial Team for the purposes of additional data analysis or for the reproducibility of this study in other centers if such is requested.

Statistical analysis was conducted using Statistical Package for the Social Sciences version 22 (Chicago, Illunois). Continuous variables are presented as mean or median values along with standard deviation, minimum, and maximum. Continuous data were compared using the independent sample *t* test for parametric data and the Mann–Whitney *U* test for nonparametric data. Categorical data were assesed using the Pearson Chi-square test, and Fisher’s exact test was used when expected value problems occurred. A value less than 0.05 was considered as statistical significant.

## Results

Among the cohorts, 37 patients in the non-radiotherapy group and 41 patients in the radiotherapy group met the inclusion criteria. There was no significant difference observed in terms of age between the groups receiving radiotherapy and those not receiving no-radiotherapy, with the mean age being 56 years in both groups. Demographic characteristics, such as body mass index, parity, vaginal delivery count, marital status, and the incidence of diabetes mellitus, exhibited comparability across both groups. Caffeine and water consumption were also similar. At least total abdominal hysterectomy and bilateral salpingo-oophorectomy was performed to the all patients included to the study. The clinical and demographic features of the patients are shown in Table 1. The vaginal length was significantly shorter in the radiotherapy group than the no-radiotherapy group (*p* < 0.012). The Q-tip test score which indicated urethral hypermobility was significantly higher in the no-radiotherapy group than the radiotherapy group. The prevalence of cystocele and rectocel yielded similar outcomes between the groups. The constituents and total score of Incontinence Severity Index in the radiotherapy and no-radiotherapy groups are depicted in Table 2. The scores on the Incontinence Impact Questionaire-7 survey, covering household chores, physical activities, social engagements, emotional well-being, and travel beyond 30 min, were significantly higher in the no-radiotherapy group than the radiotherapy group. However, frustration levels were comparable. The no-radiotherapy group demonstrated a higher total Incontinence Impact Questionnaire score compared to the radiotherapy group. Symptoms such as lower abdominal pressure, sensation of vaginal bulging, and incomplete bladder emptying showed similarity between the radiotherapy and no-radiotherapy groups, with no notable distinction. The IIQ-7 score is given on Table 3. Scores of Pelvic Organ Prolapsus Distress and Colorectal-Anal Distress Inventory were significantly higher in patients that received radiotherapy. There was no significant difference in terms of the Urogenital Distress Inventory-6 score. The Pelvic Floor Distress Inventory scores are detailed in Table 4.

**Table 1:** Clinic and demographic features of the radiotherapy group and No-adjuvant therapy groups

	No-RT	RT	*p*
Age	56.6 ± 7	56.6 ± 7	0.125
BMI	32.7 ± 4.7	33.8 ± 7.1	0.455
Parity	2.7 ± 1.2	2.3 ± 1.2	0.218
Vaginal delivery	30 (83.3)	37 (94.4)	0.106
*Menopausal status*			
Premenopausal	13 (35.1)	9 (22)	0.196
Postmenopausal	24 (64.9)	32 (78)	
*Marital status*			
Single	1 (2.7)	1 (2.4)	0.941
Married	36 (97.3)	40 (97.6)	
*DM*			
No	17 (45.9)	27 (65.9)	0.077
Yes	20 (54.1)	14 (34.1)	
*Caffein consumption*			
No	27 (73)	23 (56.1)	0.121
Yes	10 (27)	18 (43.9)	
Water consumption (cc)	1837 ± 500	1743 ± 571	0.317
*Endometrial cancer surgery*			
TAH + BSO	8 (21.6)	4 (9.8)	0.404
TAH + BSO + LND	21 (56.8)	33 (80.5)	
TLH + BSO	3 (8.1)	2 (4.9)	
TLH + BSO + LND	5 (13.5)	2 (4.9)	
*Urinary incontinence*			
None	21 (56.8)	31 (75.6)	0.078
Yes	16 (43.2)	10 (24.4)	
*Stress urinary incontinence*			
None	30 (81.1)	40 (97.6)	0.024
Yes	7 (18.9)	1(2.4)	
*Urge urinary incontinence*			
None	30 (81.1)	32 (78)	0.741
Yes	7 (18.9)	9 (41)	
*Fecal incontinence*			
None	37 (100)	38 (92.7)	0.242
Yes	0 (0)	3 (7.3)	

*RT* Radiotherapy, *DM* diabetes mellitus, *TAH* total abdominal hysterectomy, *BSO* bilateral salpingo-oophrectomy, *LND* lymph node dissection, *TLH* total laparoscopic hysterectomy, *cc* cubic centimeter

**Table 2:** ISI score of patients in radiotherapy and No-adjuvant therapy

	No-RT	RT	*p*
Vaginal length	9 ± 1.18	8.3 ± 0.23	0.012
Q-tip test	17.7 ± 11.5	12.0 ± 8.4	0.018
*Stress test*			
Negative	30 (81.1)	40 (97.6)	0.024
Positive	7 (18.9)	1(2.4)	
*Cystocele*			
None	13 (35.1)	11 (26.8)	0.108
Grade I	24 (64.9)	25 (61)	
Grade II	0 (0)	5 (12.2)	
*Rectocele*			
None	32 (85.6)	34 (82.9)	0.320
Grade I	4 (10.8)	2 (4.9)	
Grade II	1 (2.7)	5 (12.2)	
*ISI frequency*			
None	12 (32.4)	20 (48.8)	0.002
Less than once a month	9 (24.3)	13 (37.1)	
A few times a week	3 (8.1)	6 (14.6)	
Every day	9 (24.3)	0 (0)	
*ISI how much urine*			
None	12 (32.4)	20 (48.8)	0.002
Drops	9 (24.3)	18 (43.9)	
Small splashes	8 (21.6)	1 (2.4)	
More	8 (21.6)	2 (4.9)	
ISI score	3 ± 2.7	1.3 ± 1.5	< 0.001

*RT* Radiotherapy, *ISI* Incontinence Severity Index

**Table 3:** IIQ7 score of patients in radiotherapy and No-adjuvant therapygroups

	No-RT	RT	*p*
*Household chores*			
Slightly	23 (62.2)	38 (92.7)	0.002
Moderately	7 (18.9)	2 (4.9)	
Greatly	7 (18.9)	1 (22.4)	
*Physical recreation*			
Slightly	23 (62.2)	36 (87.8)	0.004
Moderately	6 (16.2)	4 (9.8)	
Greatly	8 (21.6)	1 (2.4)	
*Entertaining activities*			
Slightly	28 (75.7)	38 (92.7)	0.009
Moderately	2 (5.4)	3 (7.3)	
Greatly	7 (18.9)	0 (0)	
*Ability to travel more than 30 min*			
Slightly	24 (64.9)	36 (87.8)	0.343
Moderately	7 (18.9)	4 (9.8)	
Greatly	6 (16.2)	1 (2.4)	
*Participation in social activities*			
Slightly	31 (83.8)	34 (82.9)	0.011
Moderately	4 (10.8)	3 (7.3)	
Greatly	2 (5.4)	4 (9.8)	
*Emotional health*			
Slightly	25 (67.6)	38 (92.7)	0.004
Moderately	9 (24.3)	3 (7.3)	
Greatly	3 (8.1)	0 (0)	
*Feeling frustrated*			
Slightly	30 (81.1)	39 (95.1)	0.102
Moderately	5 (13.5)	1 (2.4)	
Greatly	2 (5.4)	1 (2.4)	
IIQ-7 score	10.7 ± 4.8	7.6 ± 1.8	0.002

Incontinence Impact Questionnaire 7, *RT* radiotherapy

**Table 4:** PFDI score of the patients in radiotherapy and No-adjuvant therapy groups

	No RT	RT	*p*
*Pressure in the lower abdomen*			
None	24 (64.9)	19 (46.3)	0.100
Yes	13 (35.9)	22 (53.7)	
*Heaviness in the lower abdomen*			
None	34 (91.9)	23 (56.1)	< 0.001
Yes	3 (8.1)	18 (43.1)	
*Feeling of bulge in vagina*			
None	35 (94.6)	37 (90.2)	0.678
Yes	2 (5.4)	4 (9.8)	
*Push vagina to complete bowel movement*			
None	30 (81.1)	7 (18.9)	0.763
Yes	7 (18.9)	6 (14.6)	
*Incomplete bladder emptying*			
None	29 (78.4)	29 (70.7)	0.604
Yes	8 (21.6)	12 (29.3)	
*Need to strain to have a bowel movement*			
None	23 (62.2)	25 (61)	1.000
Yes	14 (37.8)	16 (39)	
*Push vagina to strain to start urination*			
None	36 (97.3)	40 (97.6)	1.000
Yes	1 (2.7)	1 (2.4)	
*Incomplete bowel emptying*			
None	28 (75.7)	26 (63.4)	0.327
Yes	9 (24.3)	15 (36.6)	
*Lose stool beyond control*			
None	35 (94.6)	33 (80.5)	0.091
Yes	2 (5.4)	8 (19.5)	
*Lose gas beyond control*			
None	14 (37.8)	7 (17.1)	0.039
Yes	23 (62.2)	34 (82.9)	
*Lose liquid stool beyond control*			
None	31 (83.9)	30 (73.2)	0.257
Yes	6 (16.2)	11 (26.8)	
*Pain during defecation*			
None	32 (86.5)	32 (78)	0.332
Yes	5 (13.5)	9 (22)	
*Urgency during bowel movement*			
None	34 (91.9)	29 (70.7)	0.018
Yes	3 (8.1)	12 (29.3)	
*Lose stool during bowel movement*			
None	36 (97.3)	34 (82.9)	0.037
Yes	1 (2.7)	7 (17.1)	
*Frequent urination*			
None	15 (40.5)	11 (26.8)	0.200
Yes	22 (59.5)	30 (73.8)	
*Urgency*			
None	22 (59.5)	29 (70.7)	0.296
Yes	15 (40.5)	12 (29.3)	
*Stress urinay incontinence*			
None	30 (81.1)	40 (97.6)	0.024
Yes	7 (18.9)	1(2.4)	
*Small amount of urinary leakage*			
None	32 (86.5)	36 (87.8)	1.000
Yes	5 (13.5)	5 (12.2)	
*Difficulty in bladder emptying*			
None	34 (91.9)	35 (85.4)	0.487
Yes	3 (8.1)	6 (14.6)	
*Discomfort in genital area*			
None	31 (83.8)	33 (80.5)	0.705
Yes	6 (16.2)	8 (19.5)	
PFDI score	6.3 ± 5.7	9.6 ± 8.8	0.046
POPDI-6	1.4 ± 1.7	2.4 ± 2.6	0.041
CRADI-8	2.3 ± 2.7	3.9 ± 3.7	0.015
UDI-6	2.5 ± 2.3	3.0 ± 3.1	0.715

*RT* radiotherapy, *PFDI* pelvic floor dysfunction inventory, *POPDI-6* pelvic organ prolapse distress inventory-6, *CRADI-8* colorectal-anal distress inventory, *UDI-6* urogenital distress inventory-6

## Discussion

Treatment-associated morbidities have led clinicians to use a more conventional approach, particularly during surgery. Growing concerns regarding the morbidities associated with adjuvant therapies have heightened awareness of the quality of life among early-stage endometrial cancer patients with expected prolonged survival. In this study, we observed a decreased vaginal length in the radiotherapy group, which may have affected sexual health. Urethral hypermobility was another handicap of radiotherapy, and worse Pelvic Floor Distress Inventory scores in all subcategories of the inventory except for Urogential Distress Inventory-6 were the main findings.

Bernad et al. reported vaginal length to be 7.4 cm in endometrial cancer patients that received radiotherapy [11]. In our study, the mean vaginal length was 8.3 cm which was 9.1 in control group. Vaginal shortening was reported among the radiotherapy-induced vaginal changes and was graded 1–3 according to the level of vaginal shortening. In our study, a moderate decrease in the total vaginal length suggested that radiotherapy contributed to a grade 1 change in vaginal length. Although radiotherapy had a significant impact on reduction of vaginal length in the current sudy, the absence of Grade III changes such as vaginal stricture suggested a minimal to mild effect of radiotherapy on vagina. The combination of radiation effects on the vaginal mucosa and vaginal length may exacerbate sexual dysfunction. However, the current study lacks information on the effect of radiotherapy on sexual health. Thus, the clinical importance of shortening the vaginal length may be another field of study.

Pelvic floor abnormalities, including urinary incontinence such as urge and stress urinary incontinence, are commonly observed in women receiving treatment for endometrial carcinoma. Stress urinary incontinence has been documented to occur in up to 80% of individuals diagnosed with endometrial cancer [11]. Stress urinary incontinence was less frequent in patients who received radiotherapy. Segal et al. reported that stress urinary incontinence was comparatively less common in endometrial cancer patients who received radiotherapy compared to patients with No-adjuvant treatment, yet no statistically significant difference was observed. In addition to vaginal length shortening, increased fibrosis in the vaginal mucosa may have contributed to urethral stabilization, thereby decreasing urethral hypermobility. A significantly lower Q-tip test score observed in the radiotherapy group, potentially linked to the fibrotic effect of radiation therapy. Urethral hypermobility which was measured with dynamic MRI was reported to be as high as 47% [12]. In contrast to MRI evaluation, the Q-tip test provides more accurate functional indicators than MRI, which solely provides an anatomic assessment. As far as we know no study has evaluated the Q-tip test in endometrial cancer patients as an objective measure of urethral hypermobility. A decreased Q-tip angle may be a result of radiation therapy possibly causing fibrosis in the pelvis in endometrial cancer patients.

Pelvic organ prolapse may accompany to endometrial cancer which has been reported to be approximately with the incidence of 0.2% to 1% [13]. Although grade I cystocele and rectocele were more frequent in the no-radiotherapy group, grade II cystocele and rectocele were more frequent in the radiotherapy group. The fibrosis effect of radiotherapy may ameliorate Grade I posterior and anterior vaginal relaxation. Considering risk factors such as obesity and increased age observed in the current study for endometrial carcinoma, the increased incidence of grade I cystocele and rectocele was an expected result, and similar to urethral hypermobility, radiotherapy may have a favorable effect. Some published studies have included concomitant urogynecologic surgery in addition to surgery for endometrial cancer [14]. Although improvements in urinary incontinence were observed in these studies, none of them reported improvements in quality of life [15]. We believe that concomitant surgery for endometrial cancer patients has potential risks, including fistula formation, mesh erosion, increased operation time, and treatment failure. As a result, we do not perform urogynecologic operations during endometrial cancer surgery. A sequential approach may be more suitable for reducing anti-incontinence surgery-related risks, which may prolong the time for the application of adjuvant treatments.

Incontinence as a disease which is common among the elderly women is a condition that reduce quality of life. The severity of incontinence is associated with the reduction of quality of life. It is important to identify and manage additional conditions that may further impair the already compromised quality of life in patients undergoing long and arduous treatments for cancer. Among the scales the Incontinence Severity Index was reported to be a useful tool for evaluating quality of life and incontinence-related finding [16]. It was surprising to observe that radiotherapy, which generally lowers the quality of life, had a positive impact on urinary incontinence, another condition that impairs quality of life. Specifically, we found that the radiotherapy group had less severe urinary incontinence, as shown by the decreased scores on the Incontinence Severity Index. This suggests that radiotherapy may help reduce the severity of stress urinary incontinence. Interestingly, while the severity of urge incontinence was similar between the radiotherapy and control groups, the positive effect of radiotherapy appeared to be more significant for stress urinary incontinence. Overall, the results of this study indicate that radiotherapy may improve the severity of urinary incontinence, particularly stress incontinence, despite the general expectation that it would worsen such conditions. Lipetskai et al. also evaluated the Incontinence Impact Questionaire score in endometrial cancer patients, with the study group and control groups divided according to the type of lymphadenectomy performed [17]. The authors reported a mean score of 10, whereas in the present study, the score was 7.6 [17]. Radiotherapy is known to increase voiding dysfunctions such as urgency and bladder spasm. Enhanced stress urinary outcomes following radiotherapy represent a new finding, as prior research predominantly focused on addressing stress urinary incontinence management rather than assessing the effect of radiotherapy.

Preliminary evidence suggests that pelvic radiotherapy can detrimentally impact pelvic floor function, potentially exacerbating issues related to urinary continence and quality of life [18]. Greater discomfort due to pelvic floor dysfunction symptoms has also been associated with a worse quality of life [19]. However, the dose, route of radiotherapy, and technique used during treatment may influence the severity of radiation therapy [20]. Lakomy et al. reported similar Pelvic Floor Distress Inventory scores in endometrial cancer patients and patients with noncancerous gynecologic diseases [21]. Endometrial cancer surgery has also been shown to increase symptoms related to pelvic floor dysfunction independent of adjuvant therapies. Urinary problems are the leading problems, followed by colorectal and anal problems related to pelvic organ prolapse [22]. Both studies included patients who underwent radical hysterectomy. Urinary problems, reported as the most common pelvic floor dysfunction, may be considered the result of radical hysterectomy. In the present study, we excluded radical hysterectomy patients, which should be considered a confounder for pelvic floor dysfunction. As a result the outcomes presented in the current study may be attributed to the effect radiotherapy.

This study has several strengths. The study had criterias to eliminate any factor that would include or exclude patients in order not to interfere with pelvic floor dysfunction. Furthermore, acute effects of surgery and radiotherapy were excluded by including patients in the study at the first follow-up, which should be at least three months. This single-institution study avoided heterogeneity between institutions. Possible confounders, such as age, Body mass ındex, parity and vaginal delivery status, and menopausal status, which may affect the urinary incontinence, pelvic floor functions, and similar occurrences in the study and control groups, were considered, increasing the reliability of the outcomes related to radiotherapy. The main limitation of this study is the lack of preoperative and pre-radiotherapy baseline urogynecological evaluation of the included patients, as well as the non-application of the scoring systems used. Therefore, a study that evaluates both pre- and posttherapy periods may produce more accurate results.

Clinicians should exercise caution when considering concomitant stress urinary incontinence surgery in endometrial cancer patients undergoing endometrial cancer surgery. The observed ameliorating effect of radiotherapy on stress urinary incontinence suggests that additional surgical interventions may not be necessary and could potentially introduce unnecessary risks and complications. Given the decrease in vaginal length and potential implications for sexual function, comprehensive studies evaluating sexual health before and after radiotherapy are warranted. Incorporating preoperative and pre-radiotherapy evaluations of pelvic floor function and sexual health could provide valuable insights into the baseline status and changes induced by treatment. In addition, longitudinal studies assessing long-term outcomes beyond the immediate post-treatment period would further elucidate the durability of radiotherapy effects and inform survivorship care strategies for endometrial cancer patients.

Our study suggested that radiotherapy is associated with unfavorable pelvic floor dysfunction outcomes but may lead to better stress urinary incontinence outcomes in endometrial cancer patients.

## Data Availability

The data supporting the findings of this study are available from the corresponding author upon reasonable request.
